# Modular Sensory and Morphological Adaptations Underlying Fresh-Fruit Exploitation in Drosophilid Flies

**DOI:** 10.3390/biology15161414

**Published:** 2026-08-18

**Authors:** Shan He, Sumeng Wei, Xiaonan Yang, Guangtong Gao, Hongrun Liu, Hui Zhang, Yibo Luo

**Affiliations:** 1 College of Biosystems Engineering and Food Science, Zhejiang University, Hangzhou 310058, China; heshan1001@zju.edu.cn (S.H.); weisumengmeng@163.com (S.W.);; 2 State Key Laboratory of Plant Diversity and Specialty Crops, Institute of Botany, Chinese Academy of Sciences, Beijing 100093, China; 3 Jiaxing Future Food Research Institute, Jiaxing 314011, China; 4 College of Food Science and Nutritional Engineering, China Agricultural University, Beijing 100083, China; 5 Beijing Agricultural Technology Extension Station, Beijing 100029, China

**Keywords:** oviposition site selection, ecological overlap, modular adaptation, fermentation volatiles, mechanical barrier

## Abstract

(1) Background: Drosophilid flies oviposit in damaged or fermenting substrates, whereas *D. suzukii* prefers to lay eggs in ripening fruits. This ecological shift requires females to recognize fruit-stage cues while overcoming the mechanical barrier imposed by fresh-fruit surfaces. Whether fresh-fruit exploitation requires a complete specialist syndrome or can arise through partial combinations of sensory and morphological traits remains unclear. (2) Methods: We combined field surveys with behavioral, chemical, biomechanical and morphological analyses across multiple drosophilid species to investigate potential mechanisms underlying the diversification of fresh-fruit exploitation. (3) Results: Field surveys indicated that the phylogenetically distant species *D. immigrans* exploits fruit-associated breeding substrates that overlap with those used by *D. suzukii* in natural habitats. Females of *D. suzukii* preferred ripe strawberry cues and avoided fermentation-associated substrates, whereas *D. immigrans* showed an intermediate behavioral profile. Volatile profiling revealed that fruit decay drives chemical similarity across fruits, with rotten substrates enriched in ethyl acetate and acetic acid. Firmness measurements confirmed that decay reduces the mechanical resistance of fruit substrates, while morphological comparisons revealed divergent ovipositor architectures. (4) Conclusions: Our findings are consistent with a modular framework for fresh-fruit exploitation, in which specialist and facultative strategies reflect different combinations of fermentation-cue avoidance, substrate access, and ovipositor morphology.

## Simple Summary

Many fruit flies lay eggs in damaged or rotting plant material, but *Drosophila suzukii* can lay eggs in ripening fruit with intact skin, making it a serious pest of soft fruits. This study asked how some fruit flies can use fresh fruit while others cannot, and whether this ability depends on one special trait or on several traits working together. We compared different fruit flies in the field and in the laboratory by testing where they lay eggs, how they respond to fruit volatile organic compounds, how firm different fruits are, and the shape of their ovipositor. We found that rotting fruit becomes softer and produces similar smell signals linked to breakdown by tiny organisms such as yeast and bacteria. These signals encouraged egg laying in some fruit flies but reduced egg laying in *D. suzukii.* We also found different ovipositor shapes that may help flies interact with firmer fruit surfaces. Overall, the study shows that fresh-fruit use can evolve through different combinations of smell preference, physical access, and body structure.

## 1. Introduction

Oviposition site selection is a major determinant of insect reproductive success because females evaluate substrate quality, including nutritional resources, microbial cues, potential toxic compounds, and risks of competition or predation, thereby choosing sites that support offspring feeding, survival, and development [1,2,3]. Many herbivorous insects typically breed on fermenting or decaying fruits or other plant substrates, where yeasts and bacteria transform plant tissues into nutritionally suitable larval resources [4,5]. Microbial activity also produces volatile and contact cues, such as ethanol, acetic acid, ethyl acetate, and other yeast- or fermentation-derived compounds, that guide adult attraction, feeding, and egg-laying behavior [4,6,7,8]. Thus, for many drosophilids, fermentation-associated cues are not merely by-products of decay but reliable indicators of suitable reproductive substrates.

Within this broader ecological context, one species, *Drosophila suzukii,* represents a notable exception to the common association with damaged or decaying fruit [9]. Unlike most drosophilids, females of *D. suzukii* lay eggs at a specific fruit developmental stage: ripe fruit, but not rotten fruits [9,10]. This behavior allows larvae to develop inside fresh fruit before harvest and has contributed to the status of *D. suzukii* as a major invasive pest of soft-skinned fruit crops [11]. Beyond its economic importance, the ability of *D. suzukii* to exploit intact ripening fruit has attracted considerable attention because it is associated with coordinated changes in sensory perception, oviposition behavior, and ovipositor morphology. These linked adaptations provide a valuable model for studying how multiple trait systems evolve during shifts in reproductive niche use [12].

The transition from decaying-fruit use to fresh-fruit use imposes at least two linked challenges on ovipositing females [10,12,13]. First, females need to evaluate fruit-stage cues that change during ripening and decay. As fruits progress from ripening to rotting, their sugar composition and acidity shift, while microbial communities and volatile profiles also change [14]. Decaying substrates are often enriched in yeast- and fermentation-associated cues, including compounds such as ethyl acetate and acetic acid, which can stimulate attraction or oviposition in many drosophilids [4,6]. In contrast, fresh or ripening fruits are less dominated by fermentation-associated chemistry and require females to evaluate fruit-stage cues differently [15,16]. Second, females exploiting fresh fruits must overcome the mechanical barrier imposed by intact fruit skin and firmer tissues, which requires them to penetrate, cut, or otherwise access a more resistant oviposition substrate compared with softened decaying materials [10,12].

Previous studies have identified several traits associated with fresh-fruit use, including responses to texture and chemical cues in *D. suzukii* [12,16]. These include altered olfactory responses to fruit and fermentation cues, changes in gustatory and mechanosensory pathways, and the evolution of an enlarged serrated ovipositor capable of penetrating intact fruit skin [17,18,19,20,21]. These findings support the view that fresh-fruit use in *D. suzukii* involves coordinated sensory and biomechanical adaptation [12,18]. However, most previous comparisons have focused on *D. suzukii* and close relatives, or on contrasts between *D. suzukii* and the laboratory model *D. melanogaster* [12,13,18]. This leaves a broader evolutionary question unresolved: does fresh-fruit exploitation require a complete *D. suzukii*-like specialist syndrome, or can partial fresh-fruit use arise through different combinations of sensory, behavioral, and morphological traits?

Most previous studies on the evolution of fresh-fruit specialization have focused on *D. suzukii* and its close relatives. However, evidence that these relatives reproduce in ripe fruit under natural conditions remains limited. The closely related species *D. subpulchrella* can puncture intact ripe-fruit skin during laboratory oviposition assays [10]. A recent field study also recovered *D. immigrans*, a species generally associated with decaying substrates, from ripe strawberries [22,23]. However, its development may depend partly on prior fruit damage, and the extent to which it can independently exploit intact fresh fruit remains unclear. Because *D. immigrans* is phylogenetically distant from *D. suzukii*, it provides a useful comparison for distinguishing traits associated with facultative fresh-fruit use from those associated with strong specialization.

Here, we combined field emergence surveys, comparative oviposition assays, volatile organic compound profiling, substrate firmness measurements, and ovipositor morphological analyses across multiple drosophilid species. We asked three related questions. First, do phylogenetically distant species, especially *D. suzukii* and *D. immigrans*, overlap in natural fruit-associated breeding substrates? Second, are species differences in ripe- versus rotten-fruit oviposition associated with differential responses to yeast, sugar, and fermentation-associated volatiles? Third, are differences in fresh-fruit use accompanied by variation in substrate firmness, ovipositor architecture, and ovipositor sensilla organization? In this study, we aimed to clarify how distinct trait combinations can converge on similar ecological outcomes and thereby illuminate the diversification pathways underlying fresh-fruit use in drosophilids.

## 2. Materials and Methods

### 2.1. Experimental Design Overview

**Figure 1:**
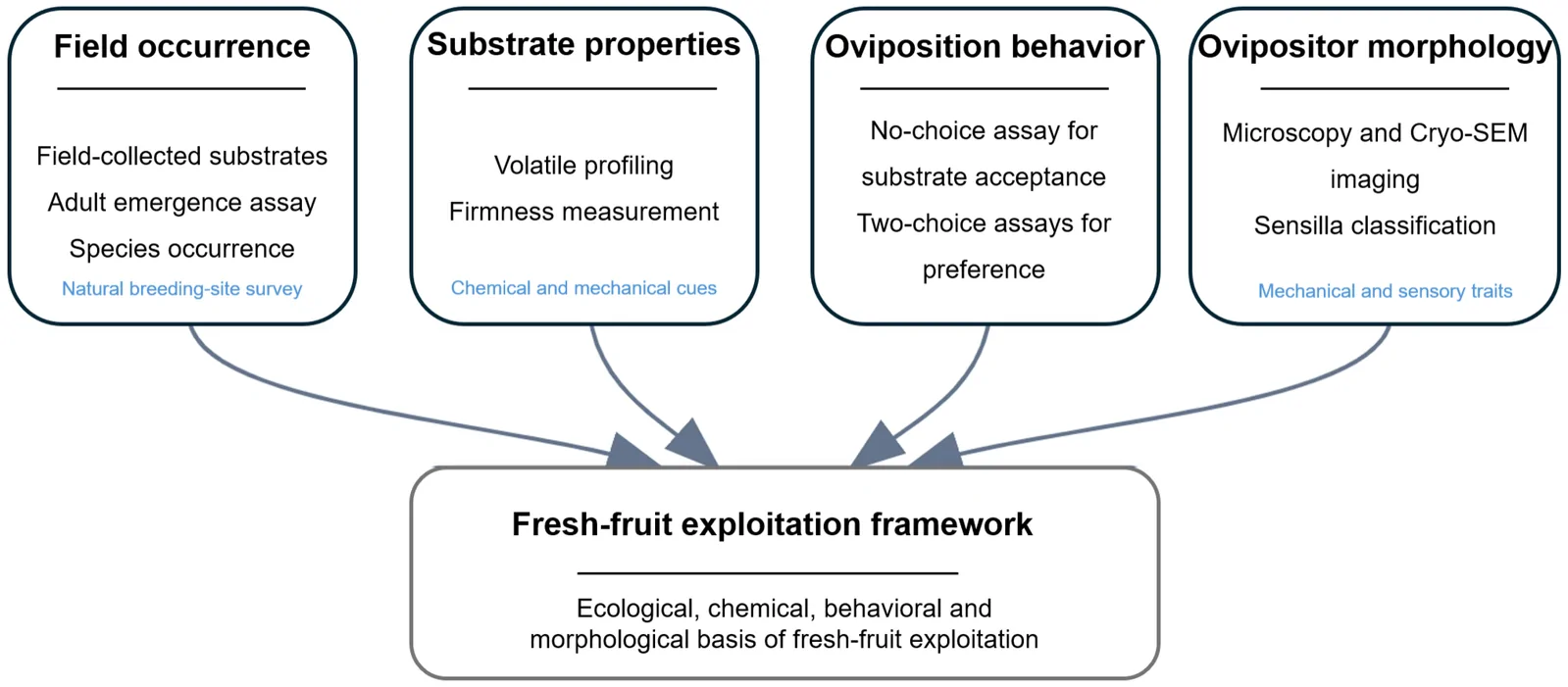
Schematic overview of the experimental design.

This study combined field emergence assays, substrate characterization, behavioral assays, and morphological analyses to evaluate whether fresh-fruit exploitation in drosophilids is associated with coordinated ecological, chemical, behavioral, and morphological traits. A schematic overview of the experimental design is provided in Figure 1.

### 2.2. Drosophila Husbandry and Strains

For standardization of behavioral and morphological experiments, a panel of wild-type strains was obtained from the Ehime Stock Center (ESC, Matsuyama, Japan), comprising *D. melanogaster* (Caton S), *D. biarmipes* (CJB214), *D. suzukii* (HJ), *D. mercatorum* (mer1527.01), *D. hydei* (DJB34), *D. virilis* (viri-HUE), *D. melanica*, *D. albomicans* (CJ56), *D. immigrans* (MT91-1), *D. simulans* (Rakujuen), *D. ananassae* (AABBg1), *D. saltans* (SHR43), and the outgroup Scaptodrosophila lebanonensis (leb0011.00). Fly stocks were maintained on a standard cornmeal–yeast–agar medium under strictly controlled environmental conditions: 24 °C, 60% relative humidity, and a 12:12 h light/dark cycle (lights on at 08:00). The standard diet consisted of 6 g agar, 7.5 g sucrose, 50 g maltodextrin, 24.5 g yeast, and 73 g cornmeal per liter of water, supplemented with 17.5 mL of methyl 4-hydroxybenzoate (10% *w*/*v* in ethanol) and 4 mL of propionic acid to inhibit microbial growth.

### 2.3. Field Collection

To identify the natural breeding sites of co-occurring drosophilids, we collected a range of plant substrates from the field (Lang Mountain region in Xinning, Hunan, China) and assayed adult emergence in the laboratory. Field collections were conducted across multiple sampling dates because different fruit species and substrates were available at different times during the sampling period. However, each substrate type was collected within a single sampling event, and samples were processed separately to minimize potential temporal variation within individual substrate categories. A total of 19 types of plant fruits were collected as substrates, including those from the genera *Citrus*, *Diospyros*, *Actinidia*, *Rubus*, and *Amygdalus*. For each substrate, 10 g was weighed and transferred into a custom emergence chamber constructed from a 10 cm diameter disposable plastic container with a lid. A 4 cm diameter aperture was cut in the center of the lid, and a layer of breathable non-woven fabric was placed between the lid and the container. Our design prevented external flies from ovipositing in the substrate while retaining newly emerged adults inside the chamber. Chambers were inspected daily. Newly emerged adults were collected with a mouth aspirator and preserved in 75% ethanol. Species were identified by combined morphological examination and DNA barcoding. Four independent biological replicates were performed for each substrate type (*n* = 4). It is important to note that flies emerging from field-collected substrates were used only to characterize substrate-associated species occurrence and were not used in the behavioral or morphological assays conducted in this study.

### 2.4. Characterization of Fruit Substrates

#### 2.4.1. Volatile Compound Collection and Identification

Headspace solid-phase microextraction coupled with gas chromatography–mass spectrometry (HS-SPME-GC-MS) was used to collect and identify volatile organic compounds (VOCs) emitted from fruits. Before sampling, individual fruit samples were placed in headspace vials. Volatiles were collected using an 85 μm CAR/PDMS SPME fiber (57334-U, Supelco-Sigma-Aldrich, St. Louis, MO, USA), with an empty vial serving as a blank control. Following extraction, the SPME fiber was manually inserted into the injection port of the gas chromatograph (GC) to desorb the analytes. We used an Agilent 7890A GC system (Agilent Technologies, Santa Clara, CA, USA) coupled with a 5975C mass spectrometry (MS) detector to identify each analyte extracted by the SPME fiber. The temperature of the GC injector was set to 280 °C, and the fiber was thermally desorbed for 1 min. The HP-5MS GC column (Agilent Technologies) was held isothermally at 40 °C for 2 min and then heated up at a rate of 12 °C/min to 300 °C and held for 3 min. The front inlet was set in splitless mode with 13.867 psi pressure, and helium was used as the carrier gas at a flow of 1 mL/min in the column. The MS source was operated at 230 °C and the MS quadrupole at 150 °C; the scanning of the MS detector was undertaken in EI mode (70 eV) and carried out from 40 to 550 at 172 scans/min. The GC–MS analysis was performed following a previously described protocol [24], without any modifications. We used commercial standard products (>95% purity; Sigma Aldrich, Saint Louis, MO, USA) of the putative compounds to compare retention time.

#### 2.4.2. Oviposition Substrate Firmness Measurements

To quantify the mechanical resistance that ovipositing females encounter, the firmness of agarose substrates across a range of concentrations and of a panel of fruits, vegetables, flaxseeds, and cacti was measured as previously described [25]. Agarose plates were prepared from a 20 mL solution in a 90 mm diameter dish (Nest, Wuxi, China). Substrate hardness was measured with a TA.HDplus Texture Analyzer (Stable Micro Systems, Godalming, UK) using a 50 mm aluminum cylinder probe with the following instrument settings: measured force in compression, pre-test speed: 1.0 mm s^−1^; test speed: 2.0 mm s^−1^; post-test speed: 10.0 mm s^−1^; strain: 50%; and trigger force: 5 g. The maximum force was used as a measure of sample firmness. All textural analyses were carried out using Texture Exponent software version 6.1.5.0 (Stable Micro Systems). The sample was positioned centrally under the probe during testing.

### 2.5. Egg-Laying Assays

#### 2.5.1. No-Choice Oviposition Assay

No-choice assays were used to determine whether females accepted a given substrate for oviposition in the absence of alternative egg-laying sites. To test acceptance of intact fruit at different ripening stages, 10 females were placed in a columnar chamber (height 10 cm, diameter 8 cm) containing a single ripe or rotten strawberry. Trials lasted 12 h in the dark (*n* = 12–19 per species), and eggs were counted by oviposition position. Ripe strawberries were purchased from a local supermarket on the day of the experiment. Ripe strawberries are defined as commercially obtained, with intact skins and no obvious physical damage. Rotten strawberries (same variety) were produced by holding fruit at 24 °C and 60% relative humidity for five days; rotten status was confirmed by visible bruising, softening, or surface discoloration before use.

To test whether individual fermentation-associated volatiles alter oviposition, 20 females were transferred into a chamber (60 mm diameter; Nest, Wuxi, China) containing a 0.5% agarose substrate supplemented with acetic acid or ethyl acetate at a final concentration of 5%, with substrates without odor supplementation serving as blank controls. The concentrations of the experimental compounds were set based on this reference [26]. After 24 h, flies were removed and eggs were counted manually under a stereomicroscope (*n* = 15).

#### 2.5.2. Two-Choice Oviposition Assay

Two-choice assays were used to test relative oviposition preference when females were allowed to choose between two substrates. Four-quadrant plastic Petri dishes (90 mm diameter; Nast, China) were used as egg-laying chambers, with opposite quadrants loaded with 5 mL agarose at the specified concentration. Unless otherwise indicated, agarose solutions were not supplemented with any other chemicals. Filled dishes were allowed to solidify for 1 h at room temperature. Twenty females were briefly cold-anaesthetized on an ice pad and immediately introduced into the chamber. After 24 h, eggs on each agarose pad were counted, and the oviposition preference index (OPI) was calculated as (eggs on experimental substrate − eggs on control substrate)/(eggs on experimental substrate + eggs on control substrate). Only assays in which females laid more than 20 eggs were recorded and analyzed, with 12–15 replicate assays per species at each concentration.

For yeast preference assays, experimental substrates were 0.5% agarose and contained 6.5% *w*/*v* yeast; control substrates were 0.5% agarose. For sucrose preference assays, experimental substrates were 0.5% agarose and contained 100 mM sucrose; control substrates were 0.5% agarose. In the strawberry puree assay, experimental substrates were filled with 35% *w*/*v* strawberry puree (ripe or rotten) in 0.5% agarose. Ripe strawberry puree was prepared by mechanically homogenizing whole ripe fruits on the day of the experiment. Rotten strawberry puree was prepared by incubating ripe strawberry puree at 24 °C and 60% relative humidity for 4–5 days before the experiment. The strawberry puree was mixed together with molten agarose cooled to approximately 45–50 °C. For the preference assay between ripe and rotten strawberry puree, experimental substrates were filled with 35% *w*/*v* ripe strawberry puree and 35% *w*/*v* rotten strawberry puree in 0.5% agarose. For the ripe or rotten strawberry puree preference assay, experimental substrates were 0.5% agarose and contained 35% *w*/*v* ripe or rotten strawberry puree; control substrates were 0.5% agarose.

### 2.6. Morphological Analysis of the Ovipositor

#### 2.6.1. Ovipositor Morphology Identification

Bright-field microscopy: Adult females were collected shortly after eclosion and euthanized at −20 °C. These were supplemented with ethanol-preserved field specimens. Ovipositors were carefully extruded and dissected from the abdomen using fine forceps under a stereomicroscope to ensure full morphological exposure. Specimens were mounted on a transparent resin base and imaged using a LEICA M205C or LEICA M165FC stereomicroscope (Leica Microsystems, Wetzlar, Germany). Scale bars (in μm) were calibrated and applied to all bright-field micrographs.

Cryogenic scanning electron microscopy (cryoSEM): Late-stage pupae were isolated from the standard medium and transferred to clean glass bottles (40–60 pupae per species). These bottles were lined with four layers of absorbent tissue paper saturated with a 5% sucrose solution to provision newly emerged adults. Following eclosion, adult flies were euthanized at −20 °C. Ovipositors were subsequently dissected and examined using a Hitachi SU8100 scanning electron microscope (Hitachi Ltd., Tokyo, Japan). Micrographs were acquired at optimal magnifications to resolve the ultrastructural details of the ovipositor sensilla.

#### 2.6.2. Sensilla Classification and Quantification

Following established morphological criteria for *Drosophila* ovipositors [10,27,28], bristles across the examined species were categorized into three primary functional morphotypes: conical peg sensilla (CP), trichoid sensilla (TS), and chaetic sensilla (CS). The conical peg sensilla were further partitioned into three distinct subtypes based on fine ultrastructural features: standard conical pegs (CP1), coeloconic conical pegs (CP2), and elongate conical pegs with finely tapered tips (CP3). Subtypes CP1 and CP2 were differentiated primarily by basal socket diameter, which is relatively smaller in CP1 and larger in CP2. In total, five bristle morphotypes were cataloged and quantified from the cryoSEM micrographs. Assuming bilateral symmetry of the terminalia, counts were performed unilaterally and reported as the number of sensilla per hemilateral plate. For each species, the number, shape, and distribution of ovipositor bristles were examined in 20 females.

### 2.7. Statistical Analysis

Statistical analyses were performed using GraphPad Prism 10.5.0. Oviposition preference indices were tested against zero using Wilcoxon signed-rank tests. Egg counts among treatments were analyzed using Kruskal–Wallis tests followed by Dunn’s multiple-comparison tests. For comparisons between two independent groups, Mann–Whitney U tests were used. Data are presented as mean ± SEM unless otherwise stated. “n.s.” indicates no significant difference; “*” indicates a significant difference (*p* < 0.05); “**” indicates a highly significant difference (*p* < 0.01); and “***” indicates a highly significant difference at *p* < 0.001. The same notation is used for all subsequent figures. The number of biological replicates (*n*) for each experiment is indicated in the corresponding Section 2 and Section 3, and figure legends. Data visualization and structural mapping were conducted utilizing GraphPad Prism 10.5.0, OriginPro 2023, Adobe Photoshop 2022, and Microsoft Office 2019.

## 3. Results

### 3.1. Phylogenetically Distant Fruit Flies Overlap in Natural Breeding Substrates

We collected fruit- and plant-associated substrates from the Lang Mountain region in Xinning, Hunan, China, and monitored adult emergence in laboratory chambers. Emerged adults were identified using morphological characters and DNA barcoding, allowing us to infer which species had successfully developed in each field-collected substrate. The field survey covered 19 substrate categories and yielded a total of 577 adults representing six drosophilid species across four replicates per substrate type.

**Figure 2:**
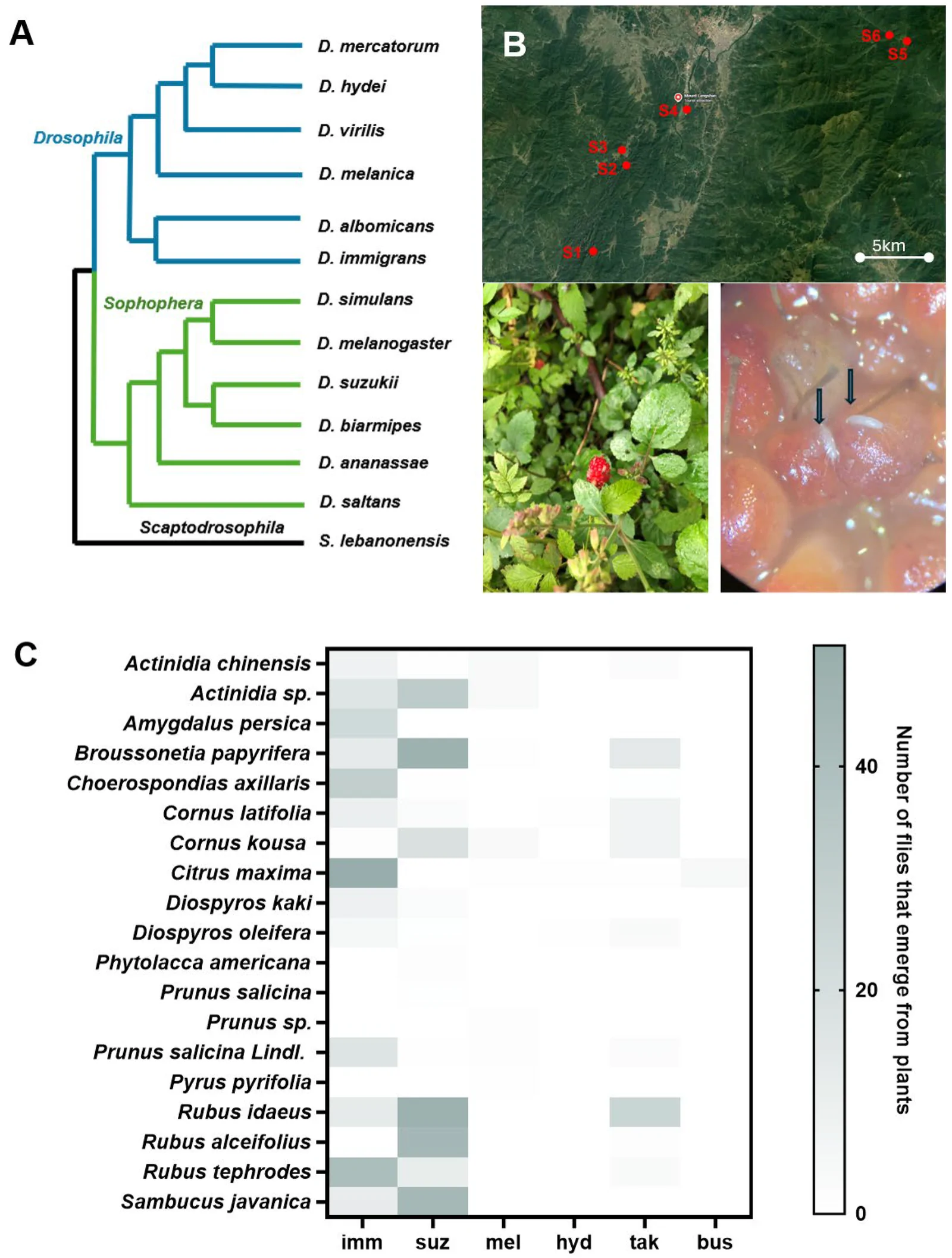
Phylogenetic context and natural breeding-site overlap among drosophilid flies. (**A**) The phylogenetic relationships and reproductive ecology of *Drosophila* species redrawn based on [29]; schematic phylogeny showing representative species within *Drosophila* (blue), *Sophophora* (green) and *Scaptodrosophila* (black). (**B**) Breeding-site survey in Xinning, Hunan, China (**top**); sampling points are marked with blue dots. Sampling sites were located at S1-26.27° N, 110.73° E; S2-26.34° N, 110.76° E; S3-26.35° N, 110.76° E; S4-26.38° N, 110.81° E; S5-26.44° N, 111.02° E; and S6-26.44° N, 111.00° E. Females of *D. immigrans* feed (**left**) and lay eggs (**right**) on the fruit of *Rubus hirsutus*; the black arrow indicates the eggs. (**C**) Number of fruit flies emerging from natural substrates on Lang Mountain. Imm-*D. immigrans*, suz-*D. suzukii*, mel-*D. melanogaster*, hyd-*D. hydei*, tak-*D. takahasii* and bus-*D. busckii*.

Substrate use was not restricted to closely related species, and the emergence data revealed substantial ecological overlap between the phylogenetically distant species *D. immigrans* and *D. suzukii* (Figure 2A). These two species were recovered from the same substrate categories in 10 of the 19 categories surveyed (Figure 2B,C). In addition, direct field observations showed *D. immigrans* adults on fresh berries, including behavior consistent with feeding and egg laying on *Rubus hirsutus* fruit (Figure 2B). These observations suggest that *D. immigrans* may associate with fresh or relatively intact fruit substrates under natural conditions. However, emergence assays cannot determine whether eggs were deposited into intact fruit or whether larvae developed after fruit damage occurred. Therefore, these data should be interpreted as evidence of ecological overlap and the potential for fresh-fruit exploitation rather than direct confirmation of intact fruit oviposition under natural field conditions.

Together, these findings show that phylogenetically distant drosophilid species can use overlapping fruit-associated breeding habitats. This ecological overlap provides a basis for testing whether *D. immigrans* and *D. suzukii* show shared or distinct behavioral, chemical, and morphological traits associated with different degrees of fresh-fruit exploitation.

### 3.2. Chemical Fruit-Stage Cues Separate D. suzukii from Fermenting-Substrate Species

**Figure 3:**
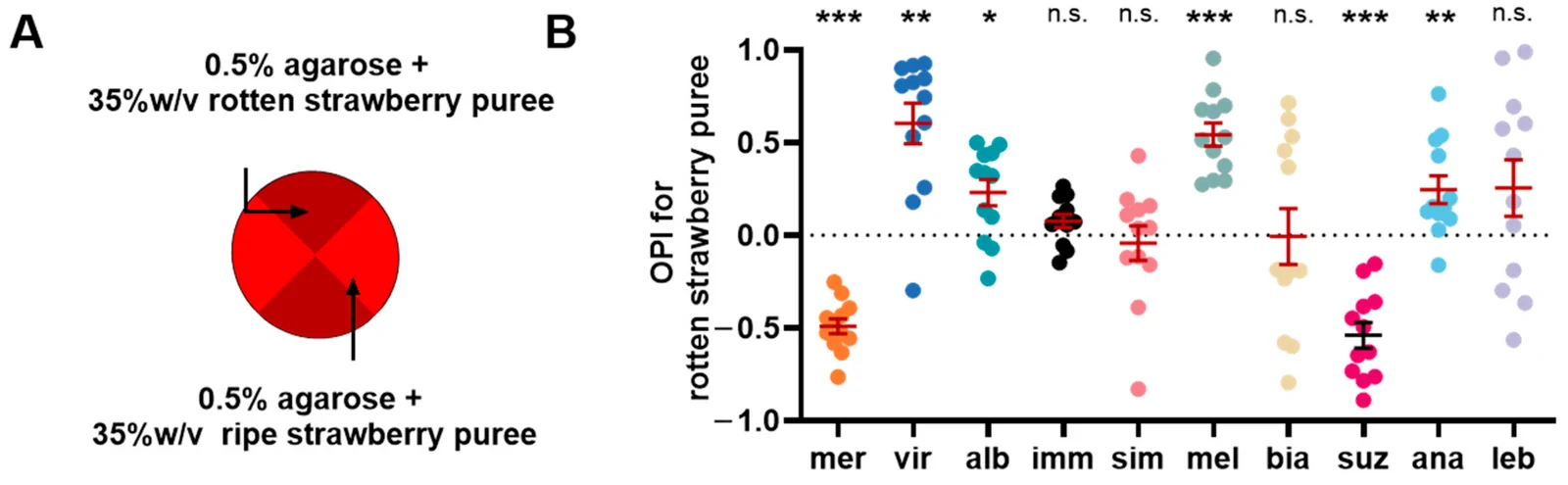
Ripe- and rotten-fruit cues separate fresh-fruit specialists from fermenting-substrate species. (**A**) Two-choice egg-laying paradigm. Twenty females chose between two substrates of equal stiffness (0.5% agarose) but different chemical composition; opposite quadrants were loaded with ripe and rotten strawberry puree prepared as described in Section 2. (**B**) Two-choice oviposition assay using ripe and rotten strawberry puree substrates. mer-*D. mercatorum*, vir-*D. virilis*, alb-*D. albomicans*, imm-*D. immigrans*, sim-*D. simulans*, mel-*D. melanogaster*, bia-*D. biarmipes*, suz-*D. suzukii*, ana-*D. ananassae* and leb-*Sca. lebanonensis*. *p* values were calculated using a Wilcoxon signed-rank test against a theoretical value of 0 (no preference). Error bars, SEM; *n* = 12. (* *p* < 0.05, ** *p* < 0.01, *** *p* < 0.001; n.s., not significant).

Fresh and rotten fruits differ in both chemical and mechanical properties, making it difficult to attribute oviposition preferences to either factor alone. To isolate the contribution of fruit-stage chemical cues, we performed a two-choice oviposition assay in a four-quadrant arena using ripe and rotten strawberry purees as standardized fruit-stage cues (Figure 3A). Each substrate consisted of ripe or rotten strawberry puree, to match the chemical stimuli of whole fruits, and equivalent concentrations of agarose (0.5%) were added to each puree to minimize mechanosensory cues. Results showed that *D. suzukii* consistently preferred substrates containing ripe strawberry puree. This result indicates that ripe-fruit chemical cues alone can support ripe-fruit-biased oviposition under mechanically standardized conditions (Figure 3B). By contrast, several other fruit flies preferred rotten strawberry puree, consistent with the widespread use of fermentation-associated substrates in this group. The lack of a strong ripe vs. rotten preference in *D. immigrans* is consistent with broader substrate acceptance, but alternative explanations, including assay-specific sensitivity, cannot be excluded without further behavioral validation (Figure 3B).

**Figure 4:**
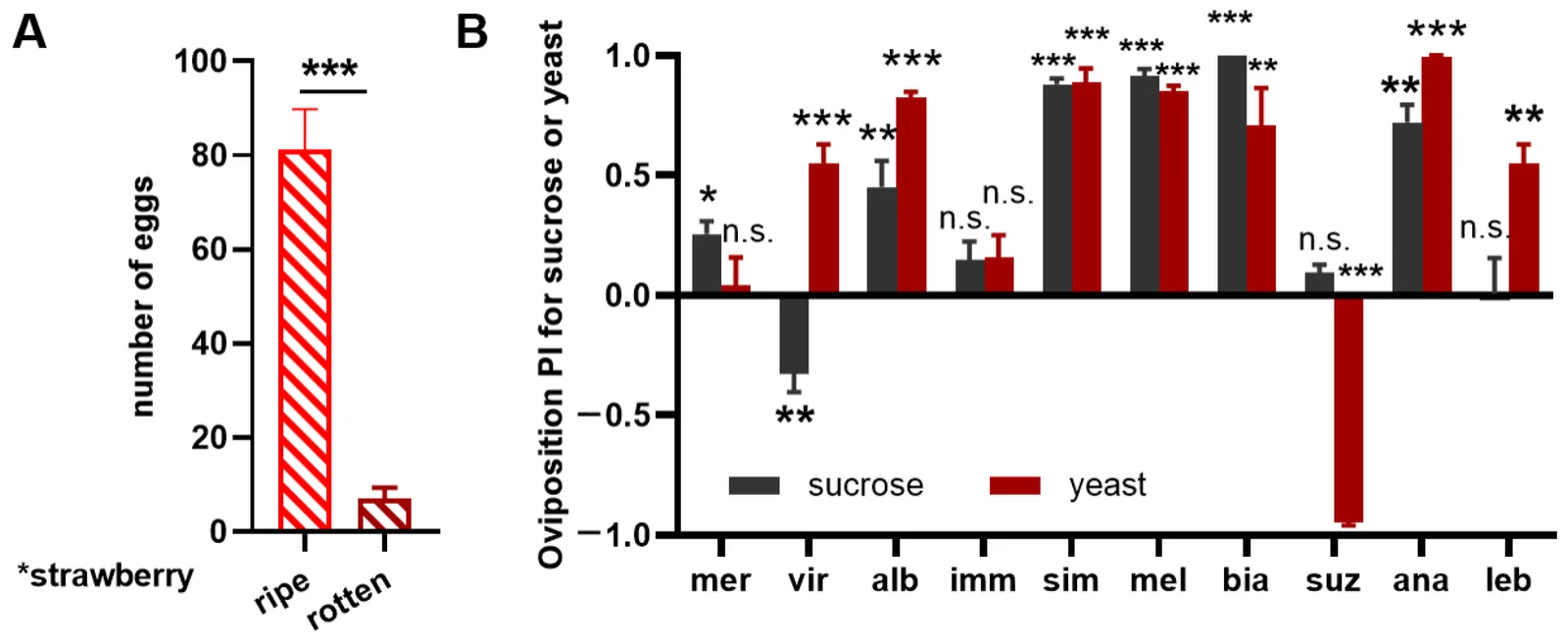
*D. suzukii* females suppress oviposition on fermentation-associated substrates. (**A**) Oviposition in *D. suzukii* was lower on rotten strawberries than on ripe strawberries. *p* values were calculated via the Mann–Whitney U test. *n* = 12. (* *p* < 0.05, *** *p* < 0.001). (**B**) Oviposition preference index (OPI) for sucrose- and yeast-containing substrates across ten drosophilid species. *p* values were calculated via Wilcoxon signed-rank tests against a theoretical value of 0. Error bars, SEM; *n* = 12. (* *p* < 0.05, ** *p* < 0.01, *** *p* < 0.001; n.s., not significant).

We then tested whether the ripe-biased response of *D. suzukii* was also evident in a whole-fruit context. In no-choice assays using an intact ripe or rotten strawberry, our results showed that *D. suzukii* deposited fewer eggs on rotten strawberries than on ripe strawberries (Figure 4A). Because no-choice assays measure substrate acceptance rather than long-range attraction, we interpret this result as reduced oviposition acceptance of rotten fruits rather than direct evidence of odor-mediated repellency.

Because fruits undergo ripening and fermentation, a multitude of metabolic processes occurs, leading to changes in chemical cues such as sugar and yeast composition [30]. We then tested whether responses to yeast or sugar were associated with the observed species differences in fruit-stage preference. In two-choice assays, females of *D. suzukii* showed no strong preference for sucrose under the tested conditions but strongly avoided yeast-containing substrates (Figure 4B). This response differed from several other drosophilid species that preferred sucrose, yeast, or both. In particular, fly species with stronger responses to rotten fruit tended to deposit more eggs on yeast-containing substrates, consistent with the role of yeast as a reproductive resource cue in many drosophilids. However, *D. immigrans* did not show the strong yeast avoidance observed in *D. suzukii*; yeast failed to elicit a significant oviposition preference in *D. immigrans* females.

These results separate the species into distinct behavioral classes. Females of *D. suzukii* showed a strong ripe-fruit-associated response, typical fermenting-substrate users preferred rotten-fruit cues, and *D. immigrans* occupied an intermediate position. The yeast assay further links the ripe-fruit preference of *D. suzukii* to its reduced acceptance of fermentation-associated substrates.

### 3.3. Fruit Decay Shifts Volatile Profiles Toward Fermentation-Associated Compounds

**Figure 5:**
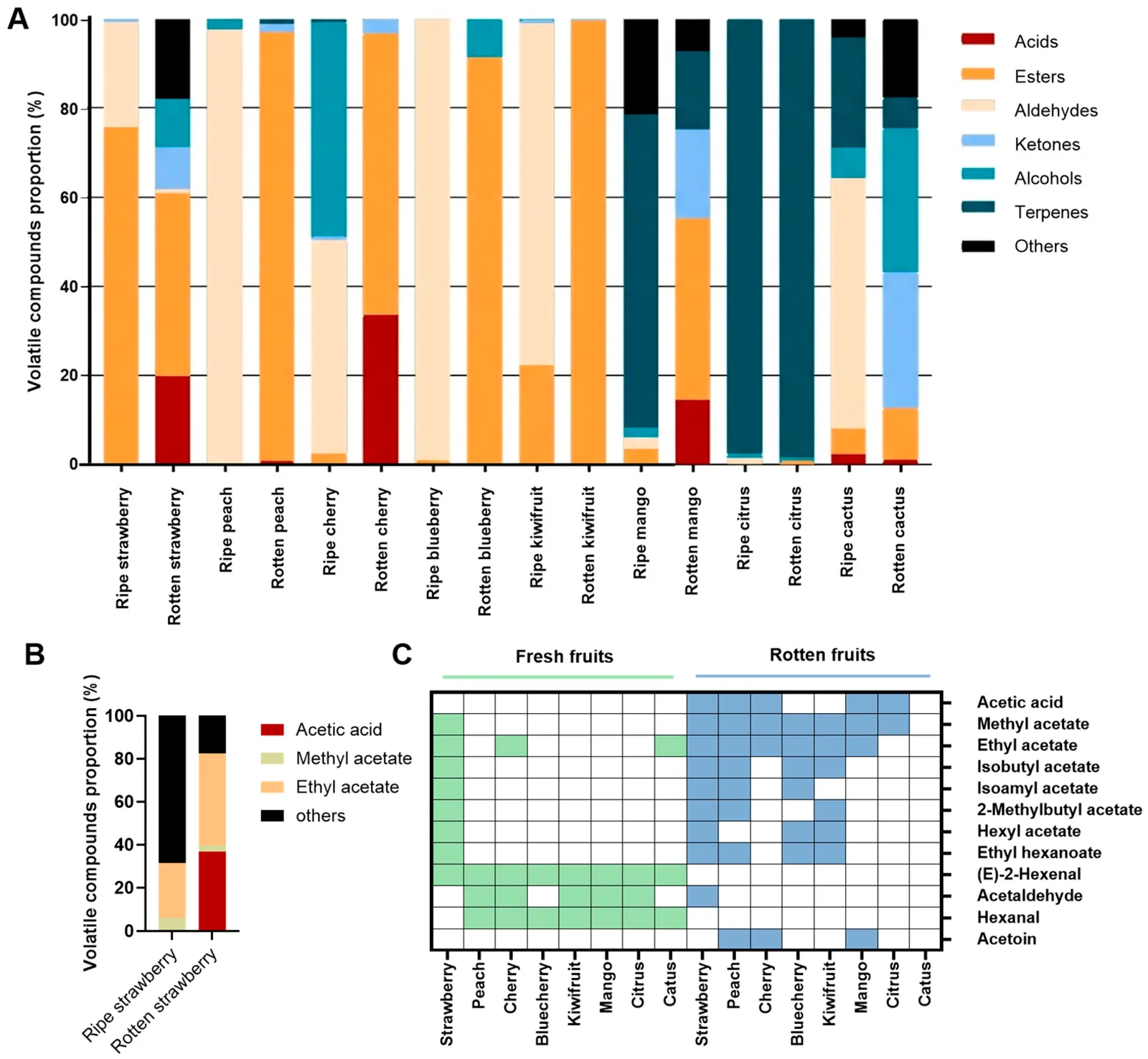
Fruit decay shifts volatile profiles toward fermentation-associated compounds. (**A**) Relative proportions of major volatile compound classes in ripe and rotten fruits measured by HS-SPME-GC-MS. Ripe fruits showed more fruit-specific volatile profiles, whereas rotten fruits showed increased representation of fermentation-associated compounds. Values represent relative peak areas. *n* = 4. (**B**) Representative volatile profiles of ripe and rotten strawberries, highlighting key fermentation-associated compounds. AA, acetic acid; MA, methyl acetate; EA, ethyl acetate. *n* = 4. (**C**) Heatmap showing the presence (colored squares) or absence (white squares) of specific volatile compounds in ripe (green) and rotten (blue) fruits. *n* = 4.

To identify candidate chemical features associated with fruit stage, we profiled VOCs emitted by ripe and rotten fruits using headspace solid-phase microextraction coupled with GC-MS. At the compound-class level, ripe samples displayed distinct fruit-specific compositions. For example, ripe strawberries were dominated by esters, ripe peaches and blueberries by aldehydes, and ripe mangoes and citrus fruits by terpenes (Figure 5A). Changes associated with decay were not uniform across all fruit types. Nevertheless, several rotten samples showed greater relative representation or more frequent detection of fermentation-associated acids and esters (Figure 5A,C). In strawberries, rotten samples showed higher relative peak-area proportions of acetic acid and ethyl acetate than ripe samples (Figure 5B). Across the broader fruit panel, the presence–absence matrix showed that selected fermentation-associated compounds, including acetic acid and ethyl acetate, were detected in a broader range of rotten- than ripe-fruit samples (Figure 5C). Together, these descriptive results indicate recurring shifts toward shared fermentation-associated volatile features during fruit decay.

Yeast-containing substrates also emitted fermentation-associated compounds that overlapped with those detected in rotten fruits, including ethyl acetate and acetic acid (Table A1). This overlap was consistent with microbial fermentation contributing to some of the volatile features observed during fruit decay. It also identified ethyl acetate and acetic acid as candidate fermentation-associated compounds for the subsequent oviposition assays.

### 3.4. Fermentation Volatiles Suppress Oviposition in D. suzukii but Stimulate Oviposition in Other Fruit Flies

**Figure 6:**
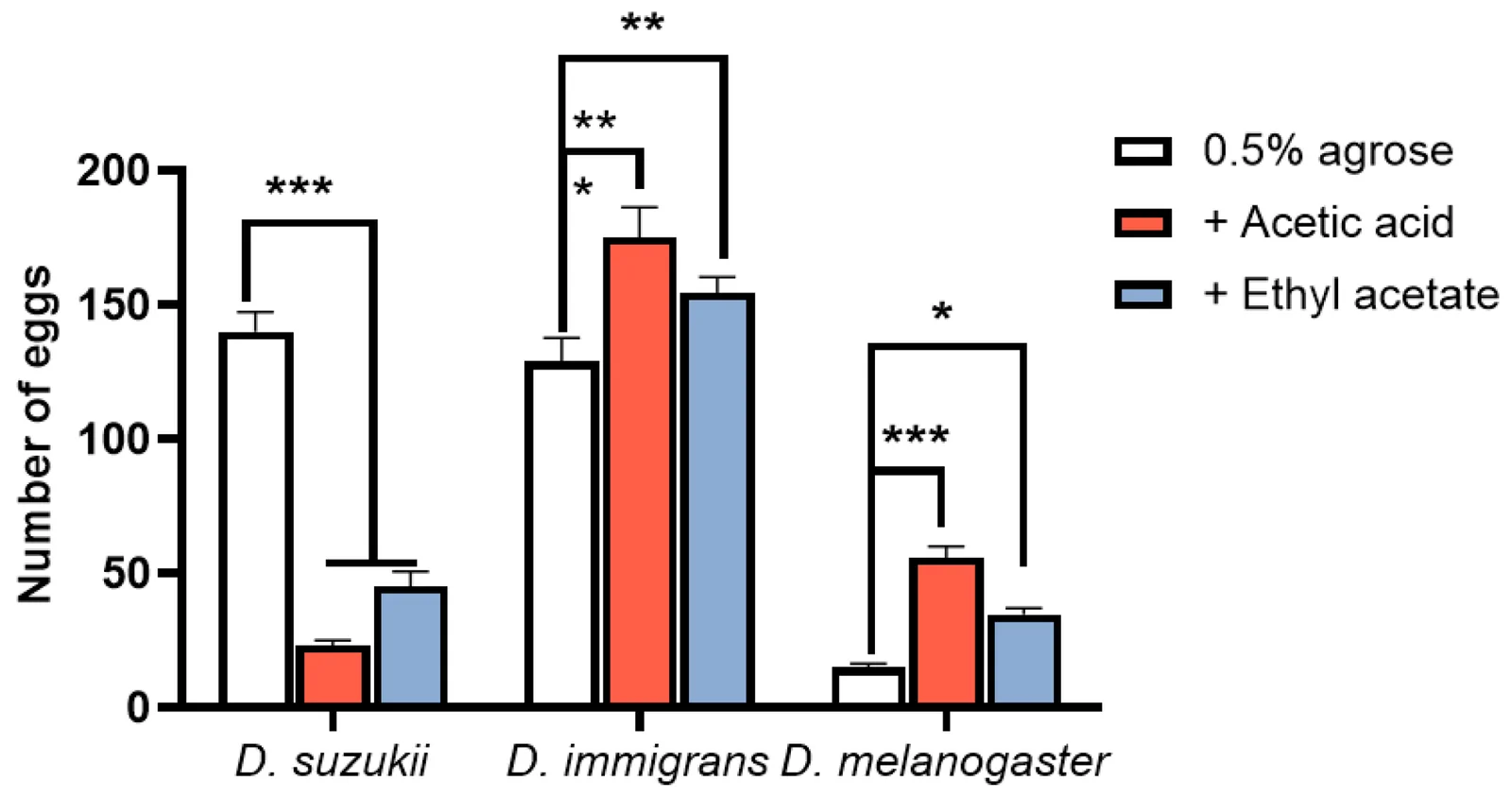
Fermentation-associated volatiles reduce oviposition in *D. suzukii* but stimulate it in other species. Oviposition responses of *D. suzukii, D. immigrans*, and *D. melanogaster* were tested in a no-choice assay in which females laid eggs on a single substrate containing 0.5% agarose alone (control) or 0.5% agarose supplemented with 5% acetic acid or 5% ethyl acetate, respectively. Females of *D. immigrans* and *D. melanogaster* laid more eggs on the supplemented substrates than on the control, whereas *D. suzukii* showed a marked reduction. *p* values were calculated using Kruskal–Wallis tests followed by Dunn’s multiple-comparison tests against the agarose control. *n* = 15 per species. Error bars, SEM. (* *p* < 0.05, ** *p* < 0.01, *** *p* < 0.001).

Having shown that decay enriches rotten fruits and yeast headspace in ethyl acetate and acetic acid, we next tested whether these two compounds could alter oviposition behavior. In no-choice assays, females of *D. suzukii*, *D. immigrans*, and *D. melanogaster* were allowed to lay eggs on control substrates or on substrates supplemented with ethyl acetate or acetic acid (Figure 6). The three species showed contrasting responses to these compounds. Females of *D. melanogaster* and *D. immigrans* deposited more eggs on substrates containing ethyl acetate or acetic acid than on control agarose. This result suggests that these fermentation-associated compounds can stimulate oviposition in both species. By contrast, *D. suzukii* laid fewer eggs on substrates containing either compound, demonstrating that the same chemical cues suppress oviposition in this fresh-fruit specialist (Figure 6).

Together, these results identify ethyl acetate and acetic acid as candidate fermentation-associated cues that differentially bias oviposition across drosophilid species. In *D. melanogaster* and *D. immigrans*, increased egg laying on compound-supplemented substrates is consistent with the interpretation that these cues can signal microbial activity or suitable larval resources. In *D. suzukii*, however, the same compounds reduced egg laying, suggesting that fermentation-associated cues may mark substrates that are less acceptable for oviposition. This contrast supports the idea that reduced acceptance of fermentation-associated compounds is one behavioral component of strong fresh-fruit specialization in *D. suzukii*, whereas *D. immigrans* shows a more flexible response rather than a *D. suzukii*-like avoidance pattern.

### 3.5. Decay Reduces Substrate Firmness and Defines the Mechanical Challenge of Fresh-Fruit Exploitation

Chemical discrimination alone cannot explain fresh-fruit exploitation because intact fruit also presents a mechanical barrier. To quantify this physical barrier, we measured the firmness of agarose substrates and a panel of plant substrates in both fresh and rotten conditions. Fresh fruits varied in firmness, with cactus showing the highest resistance and mango, citrus, kiwi, cherry, peach, and blueberry also exceeding the firmness of the low-concentration agarose substrates used in behavioral assays (Table 1).

By contrast, the decay of fruits and vegetables strongly reduced substrate firmness. Across the fruit substrates measured, rotten samples shifted toward uniformly low resistance values, approximately equal to or lower than the firmness of 0.5% agarose (Table 1). This result indicates that decay substantially reduces the mechanical barrier that ovipositing females encounter on intact or firmer substrates. Thus, rotten fruits differ from fresh fruits not only in fermentation-associated chemistry, but also in physical accessibility. These measurements provide a quantitative description of the mechanical environments associated with different oviposition substrates and fruit stages. Variation in substrate firmness may influence how readily females gain access to oviposition sites and therefore represent an important physical dimension of host use alongside chemical cues.

### 3.6. Ovipositors Show Divergent Structural Routes to Substrate Penetration and Assessment

**Figure 7:**
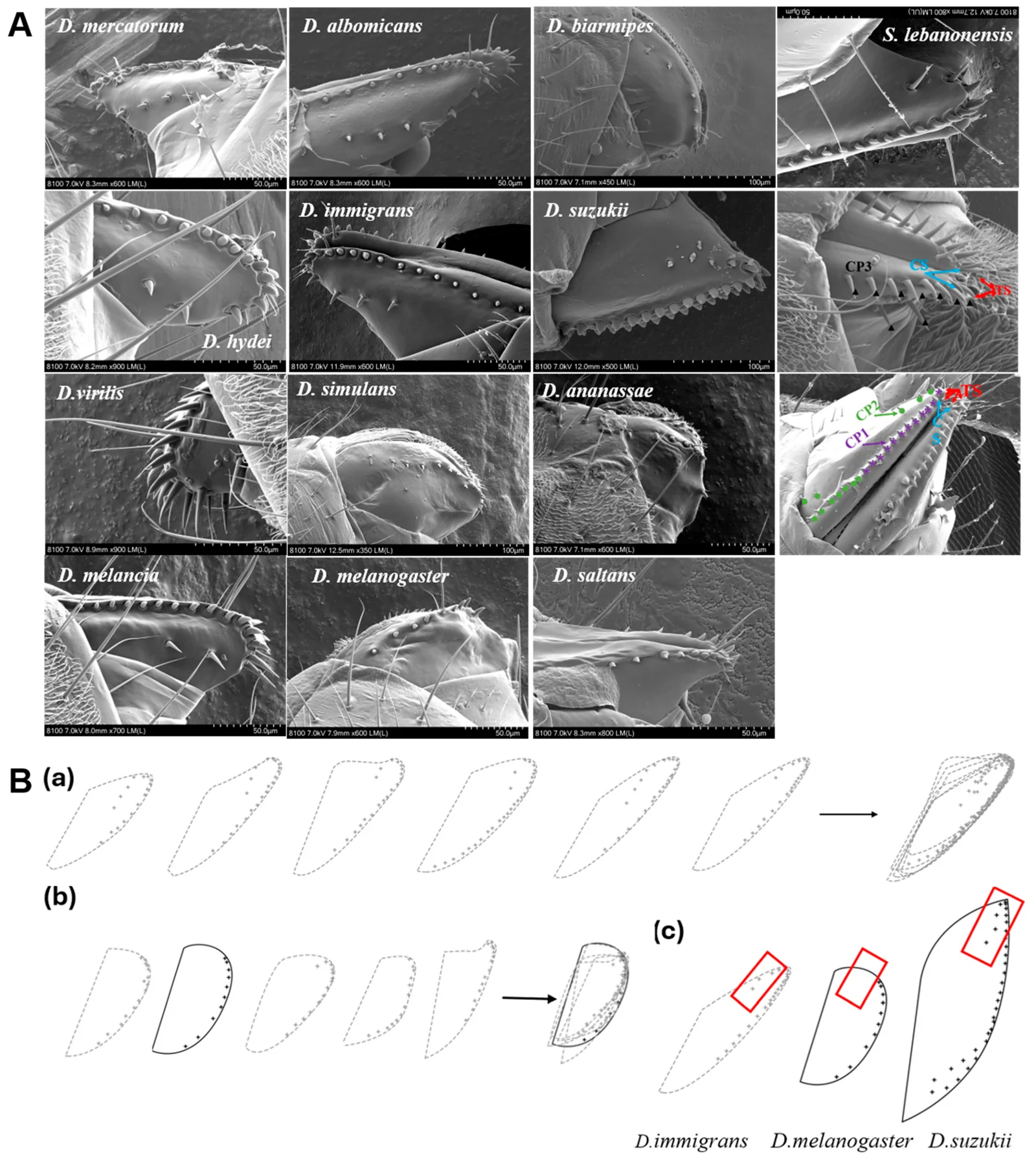
Ovipositor morphology and sensilla distribution vary across drosophilid species. (**A**) Cryogenic scanning electron micrographs of female terminalia across 13 drosophilid species. Several species in the subgenus *Drosophila*, including *D. immigrans*, displayed elongated, narrow, needle-like ovipositor plates. Most examined *Sophophora* species had shorter and blunter ovipositor plates, whereas *D. suzukii* was a distinct exception within *Sophophora*, possessing an elongated, serrated ovipositor. The outgroup species *Scaptodrosophila lebanonensis* also showed an elongated ovipositor morphology. Colored annotations indicate representative sensillum or bristle types: standard conical peg sensilla (CP1), coeloconic conical peg sensilla (CP2), elongate conical peg sensilla with tapered tips (CP3), trichoid sensilla (TS), and chaetic sensilla (CS). Scale bars (50 μm or 100 μm) are provided for each micrograph. (**B**) Schematic mapping of ovipositor plate outlines and sensilla positions. (**a**) Superimposed outlines of representative species in the subgenus *Drosophila*, showing elongated needle-like architectures. (**b**) Superimposed outlines of representative species in the subgenus *Sophophora*, showing predominantly short and blunt architectures, with *D. suzukii* as a morphological exception. (**c**) Focused comparison of *D. immigrans*, *D. melanogaster*, and *D. suzukii*. Red boxes indicate distal regions with dense sensilla or bristle distributions. Dots indicate mapped sensilla positions, and dashed outlines indicate ovipositor plate contours.

We compared ovipositor morphology across the fruit fly panel using bright-field microscopy and cryogenic scanning electron microscopy. Most examined *Sophophora* species possessed relatively short, blunt ovipositor plates, consistent with the use of softer oviposition substrates (Figure 7A). Although *D. suzukii* belongs to *Sophophora*, it was a clear morphological outlier, possessing an elongated, serrated ovipositor (Figure 7A). By contrast, several species in the subgenus *Drosophila*, including *D. immigrans*, had long, narrow, needle-like ovipositors that differed from both the blunt *Sophophora* form and the serrated ovipositor of *D. suzukii*.

We next classified and quantified ovipositor bristles and sensilla. Five morphotypes were recognized in the current analysis: standard conical peg sensilla (CP1), coeloconic conical peg sensilla (CP2), elongated conical peg sensilla with tapered tips (CP3), trichoid sensilla (TS), and chaetic sensilla (CS) (Table A2). Across species, conical peg sensilla tended to increase in size and density toward the distal end of the ovipositor plate, the region most likely to contact the substrate during oviposition. This distal enrichment is consistent with a role for the ovipositor in substrate assessment, in addition to its mechanical role during oviposition.

Our observations showed that *D. suzukii* has a particularly distinctive sensilla organization. It possessed a high total number of bristles and prominent CP1 sensilla that were not observed in the other examined species (Table A2). Several species in the subgenus *Drosophila* had more bristles than most *Sophophora* species, and some displayed CP3-type sensilla. Mapping bristle positions further showed that lateral plate bristles were present in species from the subgenus *Drosophila*, in *D. suzukii*, and in the outgroup species, but absent from many other *Sophophora* species (Figure 7B).

Together, these observations show that ovipositor traits vary across multiple structural and sensory dimensions, including plate shape, distal architecture, bristle number, sensillum type, and lateral bristle distribution. This variation appears to reflect broad phylogenetic differences, whereas the serrated ovipositor and distinctive CP1 enrichment of *D. suzukii* are consistent with a specialized condition associated with fresh-fruit oviposition. By contrast, the needle-like ovipositors observed in several *Drosophila* species, including *D. immigrans*, represent a different morphology and should not be interpreted as a direct equivalent of the *D. suzukii* ovipositor. These patterns support a modular view of ovipositor evolution, in which substrate interaction may involve different combinations of plate shape, penetration-related structures, and sensory traits across drosophilid lineages.

## 4. Discussion

### 4.1. Fresh-Fruit Exploitation Is a Modular Adaptation

Here, we use the term modular adaptation to describe a framework in which fresh-fruit exploitation can arise through partially independent trait components rather than through a single integrated specialist syndrome. Specifically, we define three modules, (i) a behavioral module involving oviposition decisions and acceptance of potential breeding substrates; (ii) a sensory module involving the evaluation of fruit-stage and fermentation-associated chemical cues; and (iii) a mechanical module involving morphological traits that influence access to fruit substrates. These modules are considered separately because each addresses a distinct ecological challenge associated with fresh-fruit exploitation and because our comparative results indicate that these traits can vary independently among species.

The evolution of ecological specialization may involve the assembly of multiple interacting traits rather than modification of a single key innovation. Previous work has emphasized the exceptional status of *D. suzukii* among drosophilid flies, particularly its evolution of a fruit-penetrating ovipositor and its ability to oviposit in ripe fruit with intact skin [12]. Early observations documented oviposition by *D. suzukii* on undamaged fruits, and later comparative studies identified adaptive modification of ovipositor morphology as an important feature associated with its use of fresh-fruit substrates [10,12]. Our results extend these observations by showing that *D. suzukii* combines this specialized ovipositor morphology with a strong behavioral bias against fermentation-associated substrates. In contrast to previous studies that primarily focused on the mechanical role of the serrated ovipositor, our comparative analyses indicate that fresh-fruit exploitation involves both morphological access and species-specific evaluation of chemical cues.

Much of the existing literature has treated *D. suzukii* as an exceptional case of fresh-fruit use among drosophilids [7,12,27]. However, fewer studies have placed this specialization within a broader comparative framework that includes species with partial or facultative access to fresh-fruit resources. By including *D. immigrans*, our study suggests a more complex landscape of fresh-fruit utilization strategies. Unlike *D. suzukii*, females of *D. immigrans* showed ecological overlap with fresh-fruit-associated habitats but only a partial combination of traits associated with fresh-fruit exploitation. This contrast is consistent with previous work suggesting that sensory and behavioral components of host or substrate use can evolve partly independently [12,18].

More importantly, our results suggest that access to fresh or relatively intact fruits and reduced acceptance of fermentation-associated cues are not necessarily coupled. Some previous interpretations of *D. suzukii* specialization have emphasized the coordinated evolution of fresh-fruit preference, fermentation-cue avoidance and ovipositor morphology [7]. Our comparative data suggested that these components may be partially decoupled across species. Females of *D. suzukii* exhibit both strong fresh-fruit-associated oviposition and reduced acceptance of fermentation-associated substrates, whereas *D. immigrans* may show only part of this combination. These findings suggest that fresh-fruit use can arise through different combinations of behavioral, sensory, and morphological traits, rather than through a single fixed specialist syndrome [12,18,31].

### 4.2. Avoidance of Chemical Cues Contributes to Fresh-Fruit Specialization

Our results indicate that oviposition behavior in D. suzukii is not determined solely by the mechanical ability to access intact fruits but also depends on altered behavioral responses to fermentation-associated chemical signals. This response contrasts with the ecology of many fermenting-substrate-associated drosophilids, in which yeast-derived volatiles, including acetic acid and ethyl acetate, commonly function as attractive or oviposition-promoting cues [4,5,32]. In our study, females of *D. melanogaster* and *D. immigrans* increased egg laying in response to these compounds, whereas *D. suzukii* showed the opposite response (Figure 6). This qualitative reversal suggests that fresh-fruit specialization involves not simply reduced sensitivity to fermentation cues, but a different interpretation of their ecological meaning during oviposition decisions. Consistent with previous studies of olfactory receptor function in *D. suzukii*, our findings support the hypothesis that changes in chemosensory processing contribute to the reduced acceptance of fermentation-associated substrates [16,20].

This sharp contrast in behavioral magnitude aligns with functional neurogenetic comparisons between *D. suzukii* and *D. melanogaster*. Specifically, while *D. melanogaster* Or85a displays high sensitivity to key fermentation esters like ethyl acetate, *D. suzukii* has experienced functional shifts and expression changes in orthologous receptors (such as Or85a and Or22a), which may alter its olfactory tuning to fermentation products and fruit-associated volatiles [33,34]. In addition, gustatory receptors and associated sensory pathways likely contribute to oviposition decisions by integrating contact cues related to substrate quality and suitability [13]. Together, these sensory modifications suggest that fresh-fruit specialization involves a reconfiguration of multiple sensory systems rather than changes in a single olfactory pathway.

Importantly, our results should not be interpreted as evidence that individual compounds such as ethyl acetate or acetic acid determine oviposition decisions in natural habitats. Natural fruits emit complex blends of volatiles, and the oviposition decisions of insects are influenced by multiple sensory modalities, including olfactory, gustatory, and mechanosensory inputs [13,26,35]. Because the concentrations used here exceed typical headspace levels of naturally rotting fruits, these assays should be interpreted as tests of behavioral sensitivity rather than naturalistic dose–response relationships. Future field experiments using intact fruits and concentration gradients will be necessary to determine how chemical and mechanical cues interact under natural conditions.

### 4.3. Ovipositor Morphology Suggests Multiple Mechanical Routes to Substrate Access

Together, our results indicate that fresh-fruit exploitation is associated with multiple partially independent trait modules. The morphological data show that ovipositor architecture varies strongly across fruit flies. The serrated ovipositor of *D. suzukii* is consistent with previous work identifying this structure as a key adaptation for laying eggs in intact fruit skin [12,19,27]. However, our comparative panel also shows that other species, particularly in the subgenus *Drosophila*, possess long needle-like ovipositors that differ from both the blunt ovipositors of many *Sophophora* species and the serrated ovipositor of *D. suzukii* (Figure 7A,B). This suggests that species may solve the mechanical challenge of substrate access in different ways.

A serrated ovipositor may facilitate cutting into intact fruit skin [10,12], whereas a needle-like ovipositor may permit puncture into softer regions, cracks, damaged surfaces, or substrates that require depth rather than cutting. These proposed functions remain hypotheses, but they help interpret why *D. immigrans* could potentially access some fresh-fruit substrates without sharing the full *D. suzukii* morphology. The comparison also cautions against treating “fresh-fruit adaptation” as synonymous with “serrated ovipositor”. A serrated ovipositor may be one specialized solution, but it is unlikely to be the only mechanical route by which fruit flies interact with firmer substrates [36]. The key implication is that fresh-fruit exploitation should not be viewed as a single *D. suzukii*-like adaptive syndrome. Instead, it may represent a modular ecological transition, in which different species gain access to similar resources through distinct combinations of mechanical, sensory, and behavioral traits [12,31,37].

Our sensilla data add another layer to this interpretation. Ovipositor bristles and conical peg sensilla are positioned where females contact the substrate during egg laying, and their distal enrichment suggests a role in substrate assessment [36,38,39]. The distinctive CP1 sensilla of *D. suzukii*, together with its increased bristle number and lateral distribution pattern (Figure 7B and Table A2), may represent a sensory specialization associated with evaluating intact fruit surfaces. Future functional studies will be required to determine how these sensilla contribute to substrate evaluation.

## 5. Conclusions

Together, our results support a modular framework of fresh-fruit exploitation in drosophilid flies. Females of *D. suzukii* combine reduced acceptance of fermentation-associated cues with a serrated ovipositor, a morphology previously associated with access to intact fruit. By contrast, the comparison with *D. immigrans* suggests that facultative or flexible use of fresh fruit may occur without the complete *D. suzukii*-like combination of traits. Fresh-fruit exploitation therefore appears to involve several components that may be partly independent, including chemical-cue evaluation, oviposition behavior, substrate accessibility, and ovipositor morphology. This framework provides a broader view of how drosophilid flies may transition from fermenting substrates toward fresh or less-decayed fruit resources. Future studies integrating broader drosophilid comparisons and field-based behavioral validation will be important for determining how widely this modular framework applies across diverse ecological contexts.

## Figures and Tables

**Table 1:** Firmness of plant substrates at fresh and rotten stages.

Substrate Type	Fresh	Rotten
cactus	506.6 ± 30.36	3.3 ± 0.8
mango	275.4 ± 17.00	2.72 ± 0.68
citrus	251.6 ± 14.4	3.14 ± 0.79
kiwi	234.6 ± 13.6	3.74 ± 1.25
cherry	212.5 ± 11	2.72 ± 0.68
peach	204.42 ± 14.05	2.6 ± 1.14
blueberry	195.5 ± 10.2	3.22 ± 0.91
vegetables	15.5 ± 2.4	2.52 ± 0.75
flower	15.56 ± 3.35	2.26 ± 1.01
0.5% agarose	4.22 ± 0.33	
1.5% agarose	65.26 ± 0.35	

*N* = 5 for each substrate. Units: ×10^4^ N/m^2^.

## Data Availability

The authors confirm that all data are available in this paper.

## Abbreviations

The following abbreviations are used in this manuscript.

CryoSEM	Cryogenic scanning electron microscopy
GC-MS	Gas chromatography–mass spectrometry
VOCs	Volatile organic compounds

## Appendix A

**Table A1:** Dynamics of various compounds in headspace volatiles of yeast.

Retention Time	Hit Name	Con
2.501	Ethanol	**
3.288	Ethyl Acetate	**
3.416	1-Propanol, 2-methyl-	**
3.684	Acetic acid	**
4.366	Propanoic acid, ethyl ester	*
4.78	1-Butanol, 3-methyl-	**
5.677	Butanoic acid, ethyl ester	*
6.633	Butanoic acid, 3-methyl-	*
6.837	1-Butanol, 3-methyl-, acetate	*
7.968	Cyclohexanone, 2-methyl-	*

“*”: volatile chromatographic peak area < 10^8^ Ab·s; “**”: 10^8^ Ab·s < volatile chromatographic peak area < 10^9^ Ab·s. Con: concentration.

**Table A2:** Types and numbers of ovipositor bristles of Drosophila.

Species	Conical Peg Sensilla (CP)			Trichoid Sensilla (TS)	Chaetic Sensilla (CS)	Total
	CP1	CP2	CP3			
Subgenus *Drosophila*						
*D. mercatorum*	0	15	1	3	1	20
*D. hydei*	0	20	1	3	1	25
*D. virilis*	0	0	20	3	1	24
*D. melanica*	0	0	27	3	1	31
*D. albomicans*	0	24	0	3	1	28
*D. immigrans*	0	24	0	3	1	28
Subgenus *Sophophora*						
*D. simulans*	0	15	0	2	1	18
*D. melanogaster*	0	12	0	3	1	16
*D. suzukii*	11	19	0	3	1	34
*D. biarmipes*	0	14	0	3	1	18
*D. ananassae*	0	11	0	2	1	14
*D. saltans*	0	14	1	3	1	19
Subgenus *Scaptodrosophila* (outgroup)						
*S. lebanonensis*	0	18	6	3	1	28
